# Regionale sozioökonomische Deprivation, sozioökonomischer Status und depressive Symptomatik: eine Mehrebenenanalyse mit Daten der Studie GEDA 2019/2020

**DOI:** 10.1007/s00103-025-04123-4

**Published:** 2025-09-09

**Authors:** Lina Wollgast, Christina Kersjes, Claudia Hövener, Niels Michalski

**Affiliations:** 1 Abteilung für Epidemiologie und Gesundheitsmonitoring, Robert Koch-Institut, Nordufer 20, 13353 Berlin, Deutschland; 2 Institut für Sozialwissenschaften, Humboldt-Universität zu Berlin, Berlin, Deutschland; 3 Fachbereich I: Soziale Arbeit, Alice Salomon Hochschule Berlin, Berlin, Deutschland

**Keywords:** Patient Health Questionnaire, Regionale Unterschiede, Soziale Determinanten der Gesundheit, Bildung, Einkommen, Patient Health Questionnaire, Regional health disparities, Social determinants of health, Educational level, Income

## Abstract

**Hintergrund:**

Die Prävalenz psychischer Erkrankungen ist in Deutschland mit der sozioökonomischen Position assoziiert. Internationale Studien zeigen zudem Zusammenhänge auf räumlicher Ebene mit regionaler sozioökonomischer Deprivation. In Deutschland sind diese räumlichen Zusammenhänge bisher nicht untersucht worden. Unklar ist auch, ob die Stärke individueller sozioökonomischer Unterschiede vom Ausmaß regionaler sozioökonomischer Deprivation abhängt.

**Methoden:**

Repräsentative Befragungsdaten der Studie Gesundheit in Deutschland Aktuell (GEDA 2019/2020-EHIS) (*N* = 21.876) werden verwendet, um die Prävalenzen depressiver Symptomatik (Patient Health Questionnaire- 8 ≥ 10) mittels kleinräumiger Schätzmethoden zu berechnen und zu visualisieren. Prävalenzen werden für Gruppen nach sozioökonomischer Position (Bildung und Einkommen) und dem Ausmaß regionaler sozioökonomischer Deprivation der Wohnregion verglichen. Multiple logistische Mehrebenenregressionsmodelle werden geschätzt, um die Gruppenunterschiede für Kontrollvariablen zu adjustieren.

**Ergebnisse:**

Die Häufigkeit depressiver Symptomatik zeigt keine systematische geografische Verbreitung über Deutschlands Stadt- und Landkreise. Das Depressionsrisiko fällt allerdings in sozioökonomisch hoch bzw. mittel deprivierten Gemeinden weitaus höher aus als in niedrig deprivierten Gemeinden (Odds Ratio = 3,29 bzw. Odds Ratio = 1,86). Zudem gibt es deutliche Bildungs- und Einkommensunterschiede zuungunsten von Personen mit niedrigerer sozioökonomischer Position. Diese Unterschiede sind in hoch deprivierten Regionen stärker ausgeprägt.

**Diskussion:**

Regionale sozioökonomische Deprivation ist auch in Deutschland ein Risikofaktor für beeinträchtigte psychische Gesundheit. Gruppen mit besonderem Versorgungsbedarf sind Personen mit niedrigem Bildungsniveau oder Einkommen in deprivierten Regionen.

**Zusatzmaterial online:**

Zusätzliche Informationen sind in der Online-Version dieses Artikels (10.1007/s00103-025-04123-4) enthalten.

## Abstract (en)

**Background:**

The prevalence of mental disorders in Germany is associated with socioeconomic position. International studies further indicate area-level correlations linking higher levels of socioeconomic deprivation with increased mental health burdens. However, these area-level associations have not yet been systematically examined in Germany and it is unclear whether socioeconomic disparities in mental health outcomes are contingent upon area-level socioeconomic deprivation.

**Methods:**

Representative data from the German Health Update survey (GEDA 2019/2020-EHIS) (*N* = 21,876) are used to estimate and visualize depressive symptoms prevalence (Patient Health Questionnaire- 8 ≥ 10) via small-area estimation. Prevalence rates are analyzed stratified by socioeconomic position (education and income) and by the degree of area-level socioeconomic deprivation in the residential municipalities. Multiple logistic multilevel regression models are employed to adjust for confounding.

**Results:**

The prevalence of depressive symptoms does not exhibit a systematic geographical distribution across German districts. However, risk of depression is significantly higher in highly (Odds Ratio = 3.29) and moderately (Odds Ratio = 1.86) socioeconomically deprived municipalities compared to low-deprivation municipalities. Additionally, there are clear educational and income disparities to the detriment of individuals with a lower socioeconomic position. These differences are more pronounced in highly deprived regions.

**Conclusion:**

Area-level socioeconomic deprivation is a key risk factor for compromised mental health in Germany. Individuals with lower education and income in deprived areas are particularly vulnerable, highlighting the need for targeted interventions and policies.

## Einleitung

Depressionen zählen zu den häufigsten und folgenschwersten psychischen Erkrankungen [1]. In den Jahren 2019/2020 berichteten 8,3 % der Erwachsenen in Deutschland eine depressive Symptomatik [2]. Laut Weltgesundheitsorganisation (WHO) sind Depressionen weltweit eine Hauptursache für krankheitsbedingte Beeinträchtigungen [1] und tragen wesentlich zur globalen Krankheitslast bei [3]. Sie beeinträchtigen die Lebensqualität und psychosoziale Funktionsfähigkeit stark. Betroffene erleben doppelt so häufig Tage mit funktionellen Einschränkungen und melden häufiger Krankheitstage [4]. Aufgrund ihrer Häufigkeit und umfassenden Krankheitsfolgen haben Depressionen und ihre Versorgung eine hohe Public-Health-Relevanz. Die statistische Erfassung von Depressionsdiagnosen setzt voraus, dass Betroffene das Gesundheitssystem kontaktieren. Sie hängt daher vom regionalen Versorgungsangebot (PsychotherapeutInnen, PsychiaterInnen, Fachkliniken) und der diagnostischen Kompetenz der HausärztInnen ab [5]. Alternativ werden repräsentative Surveys mit klinisch-diagnostischen Interviews durchgeführt [6]. Da diese sehr aufwendig sind, erfassen Gesundheitssurveys oft selbstberichtete depressive Symptome mittels speziell entwickelter Selbstbeurteilungsinstrumente [7].

Zahlreiche sozialepidemiologische Studien belegen den Zusammenhang zwischen niedriger sozioökonomischer Position und erhöhtem Depressionsrisiko für Deutschland [4, 8]. Bildung und Einkommen sind mit einem geringeren Risiko einer depressiven Symptomatik verbunden, weil sie Ressourcen bieten, die soziale Teilhabe gewährleisten [9] und Stresserleben reduzieren [10]. Sie prägen sowohl Bewältigungsmechanismen [11] als auch Gesundheitskompetenzen [12] und korrelieren mit gesundheitsförderndem Verhalten [12].

Neben individuellen sozioökonomischen Ressourcen beeinflussen auch Wohnort oder Wohnregion die psychische Gesundheit [13, 14]. Empirische Analysen kleinräumiger regionaler Unterschiede der psychischen Gesundheit in Deutschland sind selten, zeigen zum Teil aber deutliche räumliche Unterschiede etwa bei der Prävalenz depressiver Symptomatik [14] oder bei Depressionsdiagnosen [13]. Auch im Monitoring des Robert Koch-Instituts zeigen sich teils erhebliche Prävalenzunterschiede in der depressiven Symptomatik zwischen den Bundesländern [14] oder nach Stadt-Land betrachtet [4]. Regionale gesundheitliche Ungleichheiten werfen Fragen der Chancengerechtigkeit auf, wenn diese Ungleichheiten mit regionalen wirtschaftlichen und sozioökonomischen Unterschieden korrespondieren. Für andere Hocheinkommensländer wurden bereits Zusammenhänge zwischen regionaler sozioökonomischer Deprivation und psychischer Gesundheit festgestellt [15, 16], wobei Deprivation in der einschlägigen Literatur den Grad der Benachteiligung in einer Wohnregion beschreibt, der aus Ressourcenmangel, hohen sozialräumlichen Belastungen und eingeschränkten gesellschaftlichen Teilhabechancen resultiert [17]. Für Deutschland fehlen aktuelle systematische Zusammenhangsanalysen.

Ausgangspunkt für die Erklärung regionaler sozioökonomischer Ungleichheiten in der psychischen Gesundheit ist die Komposition der Bevölkerung, die sich durch die räumliche Konzentration von Personen mit ähnlicher sozioökonomischer Lage ergibt [18, 19]. Kontextuelle Einflüsse gehen darüber hinaus und fokussieren potenziell gesundheitsfördernde oder -gefährdende Eigenschaften des Wohnortes, die sich auf die bebaute Umwelt (z. B. Grünflächen, Versiegelung; [20]), Umweltrisikofaktoren (Lärmbelästigung, Luftverschmutzung; [21, 22]) und Aspekte der sozialen Umwelt wie gesellschaftliche Kohäsion (soziales Engagement, Zusammenhalt, Normen; [23–25]) oder Gewalt und Kriminalität beziehen [26, 27]. Diese Kontextmerkmale hängen wiederum oft von der sozioökonomischen Lage im Wohnumfeld ab. Personen mit höheren sozioökonomischen Ressourcen selektieren sich tendenziell in Wohngegenden mit vorteilhafter Umgebung [28], was durch das Einbringen von Ressourcen (z. B. Zeit, Informationen, Geld) die lokale Wirtschaft (Kaufkraft, Steuern, Nutzung von Kulturangeboten) positiv beeinflusst und damit sowohl die eigene als auch die Lebensqualität der Wohnregion stärkt [29]. Strukturen und individuelles Verhalten stehen so in einem Wechselverhältnis zueinander [23].

Internationale Studien zeigen zudem, dass Bildungs- und Einkommensunterschiede im Depressionsrisiko mit dem Grad der regionalen sozioökonomischen Deprivation variieren. Besonders vulnerabel sind Personen mit geringen sozioökonomischen Ressourcen, die in sozioökonomisch deprivierten Regionen leben [30, 31]. Nach der Theorie kollektiver Ressourcen profitieren Personen in niedrigen sozioökonomischen Positionen von den gesundheitsfördernden Eigenschaften der physischen und sozialen Umgebung, weil ihre individuellen Nachteile kompensiert werden können [30]. Dementgegen finden einige Studien eine stärkere gesundheitliche Benachteiligung von Personen mit niedrigem Bildungsniveau oder Einkommen in weniger deprivierten Regionen [32]. Die Interpretationen stützen sich auf die Theorie der relativen Deprivation, nach welcher Gesundheit neben der absoluten auch durch die relative sozioökonomische Position beeinflusst wird [30, 32]. Psychologische Mechanismen des „Upward Comparison“ (soziale Aufwärtsvergleiche; [30, 32]) erklären dies durch psychosozialen Stress, der auftritt, wenn Personen mit niedrigen Ressourcen in wohlhabenden Umgebungen die Normen dort anerkannter Lebensstile nicht erreichen oder aufrechterhalten können, was zu dauerhaftem Leistungsdruck, sozialer Isolation und schlechterer psychischer Gesundheit führen kann [30]. Innerhalb Deutschlands wurde bisher nicht untersucht, ob sozioökonomische Unterschiede in der psychischen Gesundheit von dem regionalen sozioökonomischen Kontext abhängen. Diese Frage ist von besonderer Public-Health-Relevanz, da Personen mit niedrigem Bildungsniveau oder Einkommen in deprivierten Regionen auch in Deutschland eine Risikogruppe mit besonders hohem Präventions- und Versorgungsbedarf darstellen könnten.

Der vorliegende Beitrag hat dementsprechend zum Ziel, den Zusammenhang zwischen regionaler sozioökonomischer Deprivation und der Prävalenz depressiver Symptomatik für Deutschland zu untersuchen und zu prüfen, ob individuelle sozioökonomische Unterschiede in der Prävalenz mit dem Ausmaß regionaler sozioökonomischer Deprivation variieren. Die Analyse verwendet Daten, die eine Übertragung der Ergebnisse auf die erwachsene Bevölkerung Deutschlands ermöglichen und beschreibende Aussagen zur sozioökonomischen Verteilung depressiver Symptomatik erlauben.

## Methoden

### Datengrundlage

Für die vorliegende Analyse wurden Individualdaten der Studie „Gesundheit in Deutschland Aktuell“ (GEDA) der Jahre 2019/2020 mit Regionaldaten des „German Index of Socioeconomic Deprivation“ (GISD) von 2019 verknüpft. Die GEDA-Studie ist eine bundesweite Querschnittsbefragung der in Deutschland lebenden Wohnbevölkerung, die seit 2008 vom Robert Koch-Institut erhoben wird. Für die 5. Folgeerhebung, GEDA 2019/2020-EHIS, wurden zwischen April 2019 und September 2020 23.001 Teilnehmende zu ihrem Gesundheitszustand und -verhalten, der Gesundheitsversorgung sowie ihrem soziodemografischen und ökonomischen Hintergrund befragt. Eine ausführliche Beschreibung der Methodik von GEDA 2019/2020-EHIS findet sich bei Allen et al. [33].

### Abhängige Variable: Depressive Symptomatik

Die depressive Symptomatik wurde mittels der 8‑Item-Version des „Patient Health Questionnaire“ (PHQ-8) erhoben [7]. Dieses Selbstbeurteilungsinstrument erfasst Vorliegen sowie Häufigkeit von 8 depressiven Symptomen, wie vermindertes Interesse oder Energieverlust, der letzten 2 Wochen gemäß den Kriterien einer Major Depressive Disorder (DSM-IV; [34]). Je nach Häufigkeit werden 0 bis 3 Punkte pro Item vergeben und aufaddiert, was zu einem Gesamtskalensummenwert zwischen 0 und 24 Punkten führt. Der gewählte Cut-off-Wert von 10 Punkten als Kriterium für eine depressive Symptomatik ist in der Literatur etabliert und bietet eine gute Kombination aus Sensitivität und Spezifizität [7].

### Zentrale unabhängige Variablen

Die regionale sozioökonomische Deprivation wurde mittels „German Index of Socioeconomic Deprivation“ (GISD) erfasst. Der Index misst das Ausmaß relativer sozioökonomischer Deprivation in den regionalen Verwaltungseinheiten Deutschland. Er basiert auf 9 räumlich aggregierten Einzelindikatoren, von denen jeweils 3 die Kerndimensionen des sozioökonomischen Status abbilden (Bildung, Beschäftigung, Einkommen; [17, 18]). Die Indikatoren umfassen beispielsweise den regionalen Anteil an Beschäftigten mit Hochschulabschluss (Bildung), die regionale Arbeitslosenquote (Beschäftigung) oder das durchschnittliche Haushaltsnettoeinkommen (Einkommen). Die Gewichtung der Einzelindikatoren innerhalb der Teildimensionen wird empirisch bestimmt. Die Teildimensionen gehen gleichgewichtet in den Gesamtindex ein, welcher Werte zwischen 0 und 1 annehmen kann. Für stratifizierte Analysen wurde der Index in Quintile eingeteilt, welche in der vorliegenden Studie in niedrige (Quintil 1), mittlere (Quintile 2–4) und hohe Deprivation (Quintil 5) kategorisiert wurden. Eine ausführliche Darstellung der Methodik des GISD findet sich bei Michalski et al. [17]. Die Kategorien des GISD wurden über die Gemeindekennziffer des Wohnortes mit den Individualdaten verknüpft.

Die sozioökonomische Position wurde einerseits über die Bildung anhand der CASMIN-Klassifikation (Comparative Analysis of Social Mobility in Industrial Nations) gemessen und in niedrige (1a–c), mittlere (2a–c) und hohe (3a–c) Bildung unterteilt [35]. Daneben wurden 3 Einkommensgruppen über eine Einteilung anhand des bedarfsgewichteten Nettoäquivalenzeinkommens (neue OECD-Skala) gebildet (niedrig „< 60 %“, mittel „60–150 %“ und hoch „> 150 %“ des Medianeinkommens; [36]).

### Kontrollvariablen

In den multivariaten Analysen wurde ein minimales Set an Kontrollvariablen verwendet. Zu diesen zählen das Geschlecht, das hier binär erfasst wurde, und das Alter, das in 5 Kategorien (18–29, 30–44, 45–59, 60–79, 80+ Jahre) gruppiert wurde. Eine Migrationsgeschichte wurde berücksichtigt, wenn das eigene oder elterliche Geburtsland nicht Deutschland ist. Das Vorliegen mindestens einer chronischen Vorerkrankung wurde aus den Angaben aus einer Liste ausgewählter Krankheiten zusammengefasst. Die Variable Wohnregion unterteilt den Wohnort nach Ost- oder Westdeutschland, mit einer Zuordnung von Berlin zu Ostdeutschland. Der siedlungsstrukturelle Kreistyp gemäß dem Bundesinstitut für Bau‑, Stadt- und Raumforschung (BBSR; [37]) unterscheidet auf Grundlage von Siedlungstyp und Bevölkerungsdichte zwischen kreisfreien Großstädten, städtischen, ländlichen und dünn besiedelten ländlichen Kreisen.

### Analysestrategie

Zunächst wurde die geografische Verteilung der Prävalenzen depressiver Symptomatik und der sozioökonomischen Deprivation auf Kreisebene kartografisch dargestellt. Dazu wurden Prävalenzen per Unit-Level-Small-Area-Estimation nach der Fayherriot-Methode geschätzt [38]. Die Aggregation auf Kreisebene war notwendig, da die Fallzahlen in den meisten Gemeinden zu gering für eine valide Schätzung waren. Die Small-Area-Estimation verbesserte die Schätzgenauigkeit durch Anpassung der Prävalenzen einerseits an den Stichprobenmittelwert und andererseits an Prävalenzwerte von Kreisen, die sich bezüglich der Hilfsinformationen ähneln [38]. Als Hilfsinformationen für die Small-Area-Estimation wurden folgende Kreisdaten aus der BBSR-Datenbank zu Indikatoren zur Raum- und Stadtentwicklung verwendet [37]: Arbeitslosenquote, Anteil der Beschäftigten mit akademischem Abschluss, Anteil der Beschäftigten ohne Berufsabschluss, durchschnittliches Haushaltsnettoeinkommen sowie der Anteil der Unter-25- bzw. Über-65-Jährigen.

Vor den Zusammenhangsanalysen wurde geprüft, ob räumliche Autokorrelation der abhängigen Variablen vorliegt. Diese wurde für die Prävalenzen depressiver Symptomatik auf Kreisebene mittels Morans I bestimmt. Das Maß erfasst regionale Häufung anhand systematischer Abweichungen vom Mittelwert der Prävalenz depressiver Symptomatik über alle Kreise, wobei positive Werte Ähnlichkeit und negative Werte Unterschiedlichkeit benachbarter Kreise anzeigen [39].

Für die Hauptanalyse wurden Prävalenzen depressiver Symptomatik stratifiziert nach dem Ausmaß regionaler sozioökonomischer Deprivation jeweils für die Bildungs- und Einkommensgruppen ermittelt. Die Unterschiede wurden anschließend in multiplen logistischen Regressionsmodellen für die Kontrollvariablen adjustiert. Um die Gemeinsamkeiten der Befragungspersonen desselben Wohnortes statistisch zu berücksichtigen, wurden dazu Mehrebenenmodelle mit Random-Intercept auf Gemeindeebene verwendet [40]. In weiteren Mehrebenenmodellen wurden Cross-Level-Interaktionen zwischen den individuellen sozioökonomischen Prädiktoren und der regionalen sozioökonomischen Deprivation eingeführt, um zu testen, ob die Stärke der sozioökonomischen Unterschiede vom Ausmaß regionaler Deprivation abhängt. Abschließend wurden die Ergebnisse der Interaktionsmodelle als adjustierte Prävalenzen grafisch dargestellt.

Alle Analysen berücksichtigen Personen, die zum Erhebungszeitpunkt 18 Jahre oder älter waren (*N* = 21.876). Sie verwenden eine Gewichtung, die das Stichprobendesign sowie das veränderte Teilnahmeverhalten während der COVID-19-Pandemie berücksichtigt und bezüglich zentraler demografischer Merkmale an die Verteilung des Mikrozensus anpasst. Die Analysen wurden mit Stata 17.0 (Stata Corp. 2021. Stata Statistische Software: Release 17, USA, College Station, TX.) sowie R 4.3.0 (R Core Team. 2023. R: A Language and Environment for Statistical Computing. Release 4.3.0, Austria, Vienna) durchgeführt. Statistische Signifikanz wurde bei einem *p*-Wert < 0,05 angenommen. Die Stichprobenzusammensetzung ist in Tab. 1 dargestellt.

**Tab. 1:** Stichprobenzusammensetzung, *N* = 21.876, GEDA 2019/2020-EHIS, GISD

Merkmale	Ausprägung	*n*	Anteile in %
Depressive Symptomatik	Ja	1295	8,5
	Nein	20.581	91,5
Geschlecht	Frauen	11.489	50,8
	Männer	10.330	49,2
	Missings	57	–
Altersgruppe	18–29	2016	16,6
	30–44	3626	22,1
	45–59	6196	26,6
	60–79	8285	26,7
	80+	1753	8,1
Migrationsgeschichte	Ja	3125	17,7
	Nein	18.651	82,3
	Missings	100	–
Chronische Vorerkrankungen	Mind. 1	16.522	73,0
	Keine	5354	27,0
Bildungsstatus – CASMIN	Geringe Bildung	4104	29,4
	Mittlere Bildung	9692	52,6
	Hohe Bildung	8098	18,0
	Missings	45	–
Bedarfsgewichtetes Nettoäquivalenzeinkommen	< 60 % des Medians (niedrig)	2924	24,3
	60–150 % des Medians (mittel)	10.497	55,4
	> 150 % des Medians (hoch)	5717	20,3
	Missings	2738	–
Wohnort	Ostdeutschland (inkl. Berlin)	5189	19,9
	Westdeutschland	16.687	80,1
Siedlungsstruktureller Kreistyp	Kreisfreie Großstädte	7557	30,2
	Städtische Kreise	8276	37,7
	Ländliche Kreise	2792	17,0
	Dünn besiedelte ländliche Kreise	2524	15,0
	Missings	727	–
Regionale sozioökonomische Deprivation	Niedrige Deprivation	6256	30,9
	Mittlere Deprivation	11.756	51,6
	Hohe Deprivation	3818	17,5
	Missings	46	–

*n* ungewichtet, Anteile in % gewichtet, *CASMIN* Klassifikationsschema des Bildungsniveaus (Comparative Analysis of Social Mobility in Industrial Nations)

## Ergebnisse

**Abb. 1:**
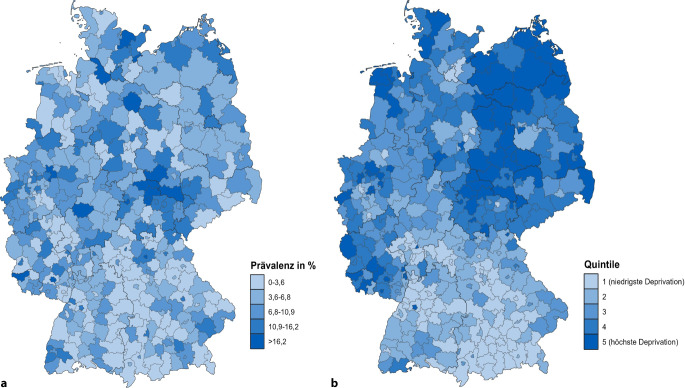
Geografische Verteilung in Stadt- und Landkreisen in Deutschland: **a** geschätzte Prävalenz depressiver Symptomatik (PHQ-8 ≥ 10) und **b** regionale sozioökonomische Deprivation, GEDA 2019/2020-EHIS, GISD. Fußnote: Die Kategorieneinteilung der geschätzten Prävalenz depressiver Symptomatik wurde anhand von Natural Breaks auf Grundlage des Jenks-Caspall-Algorithmus vorgenommen

Die Abb. 1a zeigt die geografische Verteilung der aus den Daten geschätzten Prävalenzen depressiver Symptomatik für die Stadt- und Landkreise in Deutschland. Mit Ausnahme einer räumlichen Konzentration von höheren Prävalenzen depressiver Symptomatik in und um Thüringen ist kein einheitliches regionales Muster erkennbar. Stattdessen zeigen sich benachbarte Kreise mit divergierenden Prävalenzen über das gesamte Bundesgebiet. Auffällig ist jedoch, dass sich in Großstädten vergleichsweise hohe Prävalenzen depressiver Symptomatik finden lassen. Der Eindruck einer geringen räumlichen Abhängigkeit depressiver Symptomatik wird durch den niedrigen, wenn auch signifikanten Wert für Morans I von 0,1 (*p* < 0,001) auf der Kreisebene bestätigt. Die Verteilung der regionalen sozioökonomischen Deprivation in Deutschland (Abb. 1b) weist dagegen eindeutige Muster auf. Kreise mit hoher Deprivation finden sich überwiegend in ländlichen Gebieten Nord- und Nordostdeutschlands, so in Mecklenburg-Vorpommern, Sachsen-Anhalt oder Teilen Berlin-Brandenburgs sowie in Teilen von Niedersachsen und Schleswig-Holstein. Hinzu kommen Stadt- und Landkreise, die besonders vom Strukturwandel betroffen waren [17], vor allem das Ruhrgebiet, Rheinland-Pfalz und Saarland. Regionen geringerer Deprivation sind überwiegend in Bayern, Baden-Württemberg, Südhessen und Teilen Nordrhein-Westfalens zu finden. Ein Zusammenhang zwischen den räumlichen Verteilungen depressiver Symptomatik und sozioökonomischer Deprivation lässt sich aus der augenscheinlichen Betrachtung nicht feststellen.

Die geschätzte Prävalenz depressiver Symptomatik liegt in der Gesamtstichprobe bei 8,5 % (95 % Konfidenzintervall, KI: 7,9–9,2). Stratifiziert nach sozioökonomischen Merkmalen (Tab. 2) zeigt sich eine sukzessive höhere Prävalenz depressiver Symptomatik in Gemeinden mit höherer sozioökonomischer Deprivation. Gleiche Muster zeigen sich auch in geschlechterstratifizierten Auswertungen (Tab. A2, A3 im Onlinematerial). Es können außerdem geringere Prävalenzen depressiver Symptomatik bei Personen mit jeweils höheren Bildungsabschlüssen und höheren Einkommen über alle Deprivationsniveaus hinweg beobachtet werden. Die höchsten Prävalenzen finden sich in Regionen mit hoher Deprivation vor allem bei Personen mit niedriger Bildung oder Einkommen. Punktdifferenzen der Prävalenzen zwischen Personen mit niedrigem und hohem Bildungs- bzw. Einkommensniveau sind dementsprechend am größten in regional hoch deprivierten Kontexten. Dort liegt die Prävalenz depressiver Symptomatik der niedrigsten Bildungsgruppe im Vergleich zur höchsten Bildungsgruppe um 7,4 Prozentpunkte höher (95 % KI: 2,9–11,9). Bei Personen der niedrigsten Einkommensgruppe lässt sich in hoch deprivierten Regionen sogar eine um 15,2 Prozentpunkte höhere Prävalenz (95 % KI: 8,9–21,6) im Vergleich zu Personen der höchsten Einkommensgruppe feststellen.

**Tab. 2:** Prävalenzen depressiver Symptomatik in Prozent stratifiziert nach Bildung und Nettoäquivalenzeinkommen sowie regionaler sozioökonomischer Deprivation mit 95 % Konfidenzintervallen (gewichtet), GEDA 2019/2020-EHIS, GISD

		Niedrige Deprivation (% [95 % KI])	Mittlere Deprivation (% [95 % KI])	Hohe Deprivation (% [95 % KI])
Bildung	Niedrig	9,1 [6,8–12,0]	12,2 [10,2–14,5]	12,9 [9,4–17,5]
	Mittel	7,4 [6,0–9,1]	8,1 [6,9–9,3]	11,4 [9,0–14,3]
	Hoch	4,0 [3,1–5,0]	4,2 [3,4–5,2]	5,6 [3,9–7,9]
	Gesamt	7,1 [6,1–8,3]	8,6 [7,7–9,6]	11,0 [9,2–13,1]
Nettoäquivalenzeinkommen	< 60 % des Medians (niedrig)	14,8 [11,3–19,0]	14,8 [12,3–17,7]	20,4 [15,7–26,2]
	60–150 % des Medians (mittel)	6,2 [4,9–7,7]	8,3 [7,1–9,56]	8,9 [6,8–11,6]
	> 150 % des Medians (hoch)	3,2 [2,2–4,5]	3,7 [2,7–5,1]	5,2 [2,6–10,2]
	Gesamt	7,2 [6,1–8,4]	9,0 [8,1–10,1]	11,7 [9,7–14,0]

Um die Unterschiede nach sozioökonomischer Position und GISD für soziodemografische Merkmale zu adjustieren, wurden logistische Mehrebenenmodelle geschätzt. Ein erheblicher Anteil der Variation im Auftreten depressiver Symptomatik ist auf die Unterschiede zwischen den Gemeinden zurückzuführen (Intraklassen-Korrelation = 0,486, Tab. 3 Modell 1). Dieser Wert dürfte jedoch überschätzt sein, da viele Gemeinden nur durch eine Person in der Stichprobe vertreten sind. Separate nicht ausgewiesene Berechnungen für die Kreisebene zeigten, dass etwa ein Fünftel der Variation auf Unterschiede zwischen Kreisen zurückzuführen war.

**Tab. 3:** Ergebnisse der logistischen Mehrebenenmodelle unter Angabe von Odds Ratios, 95 % Konfidenzintervallen und *p*-Werten, GEDA 2019/2020-EHIS, GISD

Merkmale	Ausprägung	(1)	(2)	(3)	(4)	(5)
Regionale sozioökonomische Deprivation (Ref. niedrige Deprivation)	Mittlere Deprivation	–	1,86^***^ [1,37;2,53]	1,74^***^ [1,27;2,38]	1,23 [0,75;2,00]	1,78^+^ [0,99;3,18]
	Hohe Deprivation	–	3,29^***^ [2,14;5,07]	2,80^***^ [1,82;4,30]	2,36^**^ [1,26;4,39]	3,50^**^ [1,44;8,52]
Bildung (Ref. hohe Bildung)	Niedrige Bildung	–	–	2,56^***^ [1,84;3,56]	1,76^*^ [1,12;2,76]	2,55^***^ [1,83;3,55]
	Mittlere Bildung	–	–	1,73^***^ [1,36;2,20]	1,52^*^ [1,00;2,31]	1,72^***^ [1,35;2,19]
Einkommen (Ref. >150 % des Medians (hoch))	<60 % des Medians (niedrig)	–	–	3,57^***^ [2,52;5,05]	3,58^***^ [2,53;5,07]	3,93^***^ [2,29;6,75]
	60–150 % des Medians (mittel)	–	–	1,86^***^ [1,39;2,49]	1,86^***^ [1,39;2,49]	1,94^**^ [1,19;3,15]
Interaktionseffekte (Ref. hohe Bildung * niedrige Deprivation)	Niedrige Bildung * Mittlere Deprivation	–	–	–	2,01^*^ [1,11;3,64]	–
	Niedrige Bildung * Hohe Deprivation	–	–	–	1,19 [0,55;2,56]	–
	Mittlere Bildung * mittlere Deprivation	–	–	–	1,21 [0,72;2,04]	–
	Mittlere Bildung * hohe Deprivation	–	–	–	1,22 [0,64;2,34]	–
Interaktionseffekte (Ref. > 150 % des Medians * niedrige Deprivation)	<60 % * mittlere Deprivation	–	–	–	–	0,85 [0,42;1,68]
	<60 % * hohe Deprivation	–	–	–	–	0,91 [0,34;2,44]
	60–150 % * mittlere Deprivation	–	–	–	–	1,05 [0,57;1,92]
	60–150 % * hohe Deprivation	–	–	–	–	0,70 [0,27;1,79]
	Random Intercept (Gemeindeebene)	3,10 [2,54;3800]	3,08 [2,50;3,79]	3,03 [2,47;3,71]	3,06 [2,49;3,75]	3,04 [2,47;3,73]
	Intraklassen-Korrelation	0,486	0,484	0,479	0,482	0,480
	*N* (Gemeinden)	2068	2068	2068	2068	2068
	*N* (Individuen)	18.362	18.362	18.362	18.362	18.362

^+^ *p* < 0,1, ^*^ *p* < 0,05, ^**^ *p* < 0,01, ^***^ *p* < 0,001

Der Gradient der regionalen sozioökonomischen Deprivation bestätigt sich in den adjustierten Ergebnissen in Tab. 3. Personen, die in regional sozioökonomisch höher deprivierten Regionen leben, weisen ein jeweils signifikant höheres Risiko einer depressiven Symptomatik auf. So liegen die Chancen (Odds) von Personen, eine depressive Symptomatik zu berichten, in mittel deprivierten Gemeinden fast doppelt so hoch wie für Personen in niedrig deprivierten Gemeinden (Modell 2 Tab. 3). Das gilt auch nach Kontrolle der individuellen sozioökonomischen Prädiktoren (Modell 3 Tab. 3). Personen in hoch deprivierten weisen im Vergleich zu solchen in niedrig deprivierten Gemeinden sogar mehr als 3‑fach erhöhte Odds für eine depressive Symptomatik auf (Modell 2 Tab. 3). Auch nach Kontrolle individueller sozioökonomischer Prädiktoren sind die Odds in hoch deprivierten Gemeinden noch mindestens doppelt so hoch (*Modell 3 *Tab. 3). Die *Modelle 4 und 5* (Tab. 3) enthalten Cross-Level-Interaktionen, die Variation in der Stärke der Bildungs- und Einkommensunterschiede je nach GISD-Kategorie zulassen. Die Spezifikation der Cross-Level-Interaktionen führt allerdings nicht zu einer signifikanten Verbesserung der jeweiligen Modelle (Ergebnisse der Wald-Tests (W): W = 6,6, *p* = 0,16; W = 3,0, *p* = 0,56). Die Modelle liefern damit keine Hinweise auf ein zusätzlich erhöhtes Risiko für sozioökonomisch benachteiligte Gruppen in regional sozioökonomisch deprivierten Regionen. Eine ausführliche Regressionstabelle findet sich im Onlinematerial (Tab. A1).

**Abb. 2:**
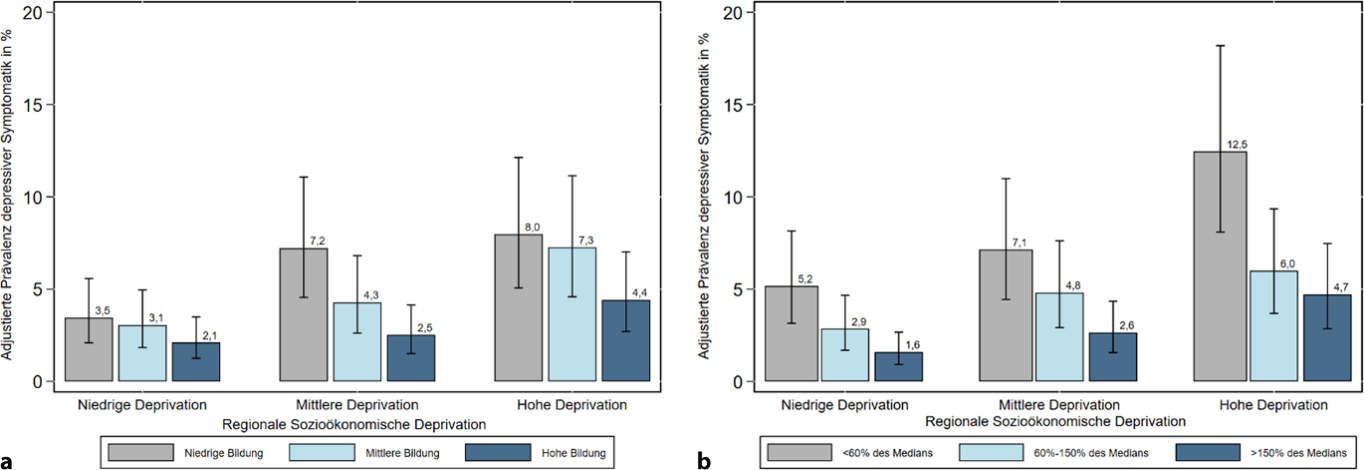
Adjustierte Prävalenzen depressiver Symptomatik in Prozent nach regionaler sozioökonomischer Deprivation und **a** formaler Bildung (gewichtet), **b** Nettoäquivalenzeinkommen (gewichtet), GEDA 2019/2020-EHIS, GISD

Werden adjustierte Prävalenzen auf Grundlage der *Modelle 4 und 5* (Tab. 3) vorhergesagt, lassen sich Bildungs- und Einkommensunterschiede im Risiko depressiver Symptomatik erkennen, die mit dem Ausmaß sozioökonomisch stärker deprivierter Gemeinden zunehmen (Abb. 2a, b). So fallen die Unterschiede in der vorhergesagten Prävalenz zwischen Personen aus niedrigen verglichen mit Personen aus hohen Bildungs- und Einkommensgruppen in hoch deprivierten Gemeinden (3,6 und 7,8 Prozentpunkte Unterschied) mehr als doppelt so hoch aus wie in niedrig deprivierten Gemeinden (1,4 und 3,6), mit besonders hohen Differenzen nach Einkommensgruppen. Die Gruppen mit der höchsten Prävalenz depressiver Symptomatik sind somit die der Personen mit niedriger Bildung (8 %) oder niedrigem Einkommen (12,5 %) in regional stark deprivierten Gemeinden.

## Diskussion

In der vorliegenden Arbeit wurden der Zusammenhang zwischen regionaler sozioökonomischer Deprivation und der Prävalenz depressiver Symptomatik in Deutschland sowie der moderierende Einfluss regionaler sozioökonomischer Deprivation auf individuelle sozioökonomische Unterschiede in der depressiven Symptomatik untersucht. Die deskriptive Betrachtung und regionalstatistische Kennwerte (Morans I) zeigten keine wesentliche räumliche Konzentration oder Dispersion der Prävalenz depressiver Symptomatik. Zudem wurden keine mit der regionalen Verteilung sozioökonomischer Deprivation korrespondierenden geografischen Muster erkannt, sodass zunächst kein Zusammenhang zwischen regionaler Deprivation und depressiver Symptomatik vermutet wurde. Die Analysen bestätigten jedoch einen Zusammenhang mit höheren Prävalenzen depressiver Symptomatik in stärker sozioökonomisch deprivierten Regionen. Der aus der internationalen Literatur bekannte Einfluss regionaler sozioökonomischer Deprivation auf das Risiko depressiver Symptomatik [15, 16] konnte damit erstmals auch für Deutschland bestätigt werden. Es zeigt sich zudem, dass der Effekt unabhängig von aggregierten individuellen sozioökonomischen Merkmalen bestand. In stärker deprivierten Kreisen traten größere Unterschiede in den Prävalenzen depressiver Symptomatik zwischen den Bildungs- und Einkommensgruppen auf. Diese höheren Differenzen waren aufgrund höherer Ausgangswerte in solchen Kreisen erwartbar. Die logistischen Regressionsmodelle erlaubten nun einen statistischen Test der Cross-Level-Interaktionen auf der Odds-Ratio-Skala und damit relativer Verhältnisse. Deren fehlende Signifikanz spricht gegen eine zusätzliche Verstärkung sozioökonomischer Unterschiede in jeweils höher deprivierten Kreisen.

Die Literatur zeigt, dass der kontextuelle Einfluss sozioökonomischer Deprivation auf die psychische Gesundheit über eine Vielzahl von Merkmalen der materiellen und sozialen Umwelt vermittelt sein kann [30]. Eine bewegungsfördernde Umgebung und Bebauung sowie insbesondere Grünflächen gehören hierbei zu den wichtigsten materiellen Umweltfaktoren [41]. Grünflächen können die psychologische Restauration durch Minderung von Stressreaktionen fördern, wodurch sie auch die Wirkung von Stressoren in höher deprivierten Regionen abschwächen können [41, 42]. Studien zeigen, dass Umweltrisikofaktoren der Luftverschmutzung und Lärmbelästigung in deprivierten Regionen häufiger auftreten [43–45] und sich negativ auf die psychische Gesundheit auswirken könnten, weil Lärmbelästigung die Schlafqualität beeinträchtigt [46] und Luftverschmutzung das zentrale Nervensystem schädigen und Inflammationen verstärken kann [47].

Im Sinne der sozialen Umweltfaktoren gilt ein geringes Ausmaß an Sozialkapital in Form von gesellschaftlicher Kohäsion und informeller sozialer Kontrolle als symptomatisch für räumliche sozioökonomische Deprivation [48]. Sozialer Zusammenhalt fördert gegenseitige Unterstützung, die sich beispielsweise in Nachbarschaftshilfen bei Reparaturen, Einkäufen oder Kinderbetreuung [25] oder im Austausch von Informationen über Jobangebote und in beruflichen Netzwerken [24] manifestiert. In solchen sozialen Kontexten können Chancen besser genutzt und Krisen besser bewältigt werden [49]. Informelle soziale Kontrolle wiederum beugt durch Eindämmung abweichenden und desintegrierenden Verhaltens Gewalt und Kriminalität vor [48], wodurch psychosozialer Stress reduziert und die psychische Gesundheit geschützt wird [50]. Einige Studien heben die „kollektive Wirksamkeit“ als wichtigste Grundlage von Sozialkapital hervor, womit die Fähigkeit der Anwohnenden gemeint ist, gemeinsame Interessen durchzusetzen [49] und so Einfluss auf die Entwicklung lokaler Bedingungen (Umwelt, Bebauung, Wirtschaft) zu nehmen [48]. Bei geringerer kollektiver Wirksamkeit wurden höhere Depressionsraten festgestellt [51].

Günstigere Lebenshaltungskosten in stärker deprivierten Regionen mit weniger vorteilhaften Umweltbedingungen führen zu einer Selektion von Personen mit geringeren sozioökonomischen Ressourcen in diese Regionen. Die kumulativen Ressourcen der Bürger vor Ort (Geld, Zeit, Informationen) haben häufig Einfluss auf Investitionen in die Infrastruktur sowohl durch das Steueraufkommen oder die Nutzung bestimmter Kulturangebote und sie prägen soziale Normen und lokale Netzwerke [29]. Kommunen in deprivierten Regionen verfügen somit häufig über weniger Mittel für gesundheitsförderliche Infrastruktur [29].

Die Analyse zeigte für Deutschland kein zusätzlich erhöhtes Risiko für Gruppen mit niedriger Bildung oder Einkommen in regional sozioökonomisch deprivierten Regionen. Aufgrund der theoretisch angenommenen gegenläufigen Wirkmechanismen – einerseits der profitablen Umwelt- und Kollektivbedingungen, andererseits psychosozialen Stresses durch sozialen Vergleich [30, 32] – und somit einer potenziellen Neutralisierung von Effekten kann keine der beiden Theorien verworfen werden.

Eine Limitation der Studie ist ihr Querschnittdesign, das es nicht erlaubt, die Vielzahl möglicher Mechanismen, die den Zusammenhang zwischen sozioökonomischen Merkmalen und depressiver Symptomatik begründen, systematisch zu testen und Kausalität nachzuweisen. Die Analysen mussten daher auf deskriptiver Ebene verbleiben. Zudem wurden die Unterschiede nur entlang der gewählten sozioökonomischen Merkmale und Kategorien betrachtet. Andere Operationalisierungen könnten zu abweichenden Ergebnissen führen. Nichtsdestotrotz weisen die Ergebnisse auf einen besonderen Versorgungs- und Förderbedarf sozioökonomisch stark deprivierter Regionen hin, in denen das Risiko depressiver Symptomatik unabhängig von individuellen sozioökonomischen Merkmalen bereits hoch ist, sodass in den niedrigen Bildungs- oder Einkommensgruppen sehr hohe Risiken erwartet werden müssen.

Eine Stärke der Analyse ist die hohe Fallzahl der repräsentativen Stichprobe, die stratifizierte Schätzungen für Bildungs- und Einkommensgruppen innerhalb regionaler sozioökonomischer Kontexte und Schlüsse auf die Bevölkerung Deutschlands ermöglichte. Dennoch sind einzelne Subgruppen, insbesondere Personen mit niedriger Bildung oder niedrigem Einkommen in stark deprivierten Regionen, nicht ausreichend stark repräsentiert, um den Vergleich von Prävalenzunterschieden statistisch abzusichern. In der Folge wurden die Analysen im Hauptteil nicht nach Geschlechtern getrennt. Ergebnisse weisen gleichwohl keine wesentlich abweichenden Koeffizienten auf (siehe Tab. A2, A3, Abb. A1, A2 im Onlinematerial).

Ein weiterer Vorteil der Daten ist die präzise Zuordnung des Wohnortes auf Gemeindeebene, wodurch über 2000 Einheiten auf der Kontextebene mit mindestens einer Beobachtung repräsentiert wurden. Prävalenzschätzungen auf Gemeindeebene sind jedoch damit nicht möglich und selbst auf Kreisebene sind die Schätzungen mit Unsicherheit und starken Annahmen verbunden. Die Gruppierung der Wohnorte nach Deprivationsniveau auf Gemeindeebene erlaubte dennoch eine vergleichsweise genaue Zuordnung der regionalen sozioökonomischen Bedingungen. Unterschiede innerhalb von Gemeinden werden mit den Daten nicht erfasst. Andererseits ließe sich schlussfolgern, dass die Assoziationen zwischen der räumlichen sozioökonomischen Deprivation und dem Risiko depressiver Symptomatik wegen der grobgliedrigen Erfassung der Deprivation am Wohnort sogar unterschätzt wurden. Die gefundenen Muster über Subgruppen und nach Kontrolle für wesentliche Kovariaten sind gleichwohl stabil und konsistent mit Ergebnissen der Literatur.

Depressive Symptomatik variiert in Deutschland zwar geografisch nicht systematisch, hängt aber von kontextuellen Faktoren ab. Regionale sozioökonomische Deprivation ist auch in Deutschland ein Risikofaktor depressiver Symptomatik. Die Ergebnisse unterstreichen die Bedeutung einer sozialraumorientierten und zielgruppengerechten Bedarfsplanung. Diese kann dazu beitragen, gesundheitliche Chancengleichheit und die Herstellung gleichwertiger Lebensverhältnisse zu fördern. Die Ergebnisse sind ein Beispiel für regionale gesundheitliche Ungleichheiten, deren Interesse in Hinblick auf bestehende Ausgleichsmechanismen, wie die Regionalkomponente im Risikostrukturausgleich der gesetzlichen Krankenversicherung [52], kritisch zu prüfen ist. Insgesamt liefern die Befunde Impulse für eine integrierte Gesundheits- und Sozialpolitik, die sowohl die strukturellen als auch die individuellen Determinanten psychischer Gesundheit in den Fokus rückt.

Für diesen Beitrag wurden von den Autorinnen und Autoren keine Studien an Menschen oder Tieren durchgeführt. Für die aufgeführten Studien gelten die jeweils dort angegebenen ethischen Richtlinien.

## Supplementary Information

Das Onlinematerial enthält ergänzende Ergebnisse zur Studie, unter anderem die auf Kreisebene geschätzten Prävalenzen depressiver Symptomatik, getrennt nach Geschlecht. Darüber hinaus werden die Ergebnisse der Mehrebenenregressionen mit vollständigen Kovariaten dargestellt, sowohl für die Gesamtstichprobe als auch getrennt nach Geschlecht.

## Data Availability

Die in diesem Beitrag verwendeten Daten der GEDA-Studie können über das Forschungsdatenzentrum des Robert Koch-Instituts bezogen werden: https://www.rki.de/DE/Aktuelles/Publikationen/Forschungsdaten/FDZ/Datenangebot/GEDA/GEDA-Wellen.html. Die Daten des German Index of Socioeconomic Deprivation sind über das Datenrepositorium des Robert Koch-Instituts frei verfügbar: https://robert-koch-institut.github.io/German_Index_of_Socioeconomic_Deprivation_GISD/. Für die Verwendung der GEDA-Daten inklusive der amtlichen Regionalschlüssel der Wohnortgemeinden, die erforderlich sind, um den GISD zu verknüpfen, gelten Zugangsbeschränkungen. Diese setzen einen GastwissenschaftlerInnenarbeitsplatz am Robert Koch-Institut voraus. Die für die Analysen verwendete Syntax kann zu Replikationszwecken auf Nachfrage bei den AutorInnen angefordert werden.

## References

[1] World Health Organization (2017) Depression and other common mental disorders. In: World Health Organization (Hrsg) Global health estimates. World Health Organization, Geneva (https://iris.who.int/bitstream/handle/10665/254610/WHO-MSD-MER-2017.2-eng.pdf?sequence=1. Zugegriffen: 16.01.2025)

[2] Hapke U, Kersjes C, Hoebel J, Kuhnert R, Eicher S, Damerow S (2022) Depressive symptoms in the general population before and in the first year of the COVID-19 pandemic: results of the GEDA 2019/2020 study. J Health Monit 7:3. 10.25646/10664 36654684 10.25646/10664 PMC9838134

[3] Ferrari AJ, Charlson FJ, Norman RE et al (2013) Burden of depressive disorders by country, sex, age, and year: findings from the global burden of disease study 2010. PLoS Med 10:e1001547. 10.1371/journal.pmed.1001547 24223526 10.1371/journal.pmed.1001547 PMC3818162

[4] Maske UE, Buttery AK, Beesdo-Baum K, Riedel-Heller S, Hapke U, Busch MA (2016) Prevalence and correlates of DSM-IV-TR major depressive disorder, self-reported diagnosed depression and current depressive symptoms among adults in Germany. J Affect Disord 190:167–177. 10.1016/j.jad.2015.10.006 26519637 10.1016/j.jad.2015.10.006

[5] Jacobi F, Becker M, Bretschneider J et al (2016) Ambulante fachärztliche Versorgung psychischer Störungen. Nervenarzt 87:1–9. 10.1007/s00115-016-0147-4 10.1007/s00115-016-0147-4 27357454

[6] Kessler RC, Wittchen HU, Abelson JM et al (1998) Methodological studies of the Composite International Diagnostic Interview (CIDI) in the US national comorbidity survey (NCS). Int J Methods Psychiatr Res 7:33–55. 10.1002/mpr.33

[7] Kroenke K, Strine TW, Spitzer RL, Williams JB, Berry JT, Mokdad AH (2009) The PHQ‑8 as a measure of current depression in the general population. J Affect Disord 114:163–173. 10.1016/j.jad.2008.06.026 18752852 10.1016/j.jad.2008.06.026

[8] Cohrdes C, Hapke U, Nübel J, Thom J (2022) Erkennen-Bewerten-Handeln. Schwerpunktbericht zur psychischen Gesundheit der Bevölkerung in Deutschland. Teil 1 – Erwachsenenalter. In: Robert Koch-Institut. https://edoc.rki.de/bitstream/handle/176904/9259/EBH_Bericht_Psyschiche_Gesundheit.pdf?sequence=1&isAllowed=y. Zugegriffen: 16. Jan. 2025

[9] Lindström M, Hanson BS, Östergren P‑O (2001) Socioeconomic differences in leisure-time physical activity: the role of social participation and social capital in shaping health related behaviour. Soc Sci Med 52:441–451. https://doi.org/10.1016/S0277–9536(00)00153‑2 11330778 10.1016/s0277-9536(00)00153-2

[10] Krieger N (2001) Theories for social epidemiology in the 21st century: an ecosocial perspective. Int J Epidemiol Public Health Res 30:668–677. 10.1093/ije/30.4.668 10.1093/ije/30.4.668 11511581

[11] Lövdén M, Fratiglioni L, Glymour MM, Lindenberger U, Tucker-Drob EM (2020) Education and cognitive functioning across the life span. Psychol Sci Public Interest 21:6–41. 10.1177/1529100620920 32772803 10.1177/1529100620920576 PMC7425377

[12] Petrovic D, de Mestral C, Bochud M et al (2018) The contribution of health behaviors to socioeconomic inequalities in health: a systematic review. Prev Med 113:15–31. 10.1016/j.ypmed.2018.05.003 29752959 10.1016/j.ypmed.2018.05.003

[13] Wissenschaftliches Institut der AOK (WIdO) (2021) Depression – Anteil erkrankter Menschen (1-Jahres-Prävalenz) in Prozent – Alle Landkreise im Vergleich. In: Gesundheitsatlas Deutschland. https://www.gesundheitsatlas-deutschland.de/erkrankung/depressionen. Zugegriffen: 9. Jan. 2024

[14] Robert-Koch Institut (2022) Dashboard zu Gesundheit in Deutschland aktuell – GEDA 2019/2020. In: Robert-Koch Institut (ed). https://public.tableau.com/app/profile/robert.koch.institut/viz/Gesundheit_in_Deutschland_aktuell/GEDA_20192020-EHIS. Zugegriffen: 31. Jan. 2024

[15] Cohen-Cline H, Beresford SA, Barrington WE, Matsueda RL, Wakefield J, Duncan GE (2018) Associations between neighbourhood characteristics and depression: a twin study. J Epidemiol Community Health 72:202–207. 10.1136/jech-2017-209453 29273630 10.1136/jech-2017-209453 PMC6007871

[16] Dowdall N, Ward CL, Lund C (2017) The association between neighbourhood-level deprivation and depression: evidence from the south african national income dynamics study. BMC Psychiatry 17:1–10. 10.1186/s12888-017-1561-2 29228912 10.1186/s12888-017-1561-2 PMC5725901

[17] Michalski N, Reis M, Tetzlaff F et al (2022) German Index of Socioeconomic Deprivation (GISD): revision, update and applications. J Health Monit 7:2. 10.25646/10641 36628258 10.25646/10641 PMC9768633

[18] Kroll LE, Schumann M, Hoebel J, Lampert T (2017) Regional health differences—developing a socioeconomic deprivation index for Germany. J Health Monit 2:98. 10.17886/RKI-GBE-2017-048.2 37152089 10.17886/RKI-GBE-2017-048.2 PMC10161274

[19] K‑b MKH‑J, Kim H‑J, J‑y M (2017) Parks and green areas and the risk for depression and suicidal indicators. Int J Public Health 62:647–656. 10.1007/s00038-017-0958-5 28337512 10.1007/s00038-017-0958-5

[20] Houlden V, Weich S, Porto de Albuquerque J, Jarvis S, Rees K (2018) The relationship between greenspace and the mental wellbeing of adults: a systematic review. PLoS ONE 13:e203000. 10.1371/journal.pone.0203000 30208073 10.1371/journal.pone.0203000 PMC6135392

[21] Petrowski K, Bührer S, Strauß B, Decker O, Brähler E (2021) Examining air pollution (PM10), mental health and well-being in a representative German sample. Sci Rep 11:18436. 10.1038/s41598-021-93773-w 34531408 10.1038/s41598-021-93773-w PMC8445943

[22] Hegewald J, Schubert M, Freiberg A et al (2020) Traffic noise and mental health: a systematic review and meta-analysis. Int J Environ Res Public Health 17:6175. 10.3390/ijerph17176175 32854453 10.3390/ijerph17176175 PMC7503511

[23] X‑MT S (2004) Investigating the relationship between neighbourhood social cohesion, economic deprivation and residents’ health. University of London, University College London, United Kingdom

[24] Stafford M, De Silva M, Stansfeld S, Marmot M (2008) Neighbourhood social capital and common mental disorder: testing the link in a general population sample. Health Place 14:394–405. 10.1016/j.healthplace.2007.08.006 17919964 10.1016/j.healthplace.2007.08.006

[25] Fone D, Dunstan F, Lloyd K, Williams G, Watkins J, Palmer S (2007) Does social cohesion modify the association between area income deprivation and mental health? A multilevel analysis. Int J Epidemiol 36:338–345. 10.1093/ije/dym004 17329315 10.1093/ije/dym004

[26] Kim J (2010) Neighborhood disadvantage and mental health: the role of neighborhood disorder and social relationships. Soc Sci Res 39:260–271. 10.1016/j.ssresearch.2009.08.007

[27] Curry A, Latkin C, Davey-Rothwell M (2008) Pathways to depression: The impact of neighborhood violent crime on inner-city residents in Baltimore, Maryland, USA. Soc Sci Med 67:23–30. 10.1016/j.socscimed.2008.03.007 18396366 10.1016/j.socscimed.2008.03.007 PMC2684449

[28] Norman P, Boyle P, Rees P (2005) Selective migration, health and deprivation: a longitudinal analysis. Soc Sci Med 60:2755–2771. 10.1016/j.socscimed.2004.11.008 15820585 10.1016/j.socscimed.2004.11.008

[29] Franzini L, Caughy M, Spears W, Esquer MEF (2005) Neighborhood economic conditions, social processes, and self-rated health in low-income neighborhoods in Texas: a multilevel latent variables model. Soc Sci Med 61:1135–1150. 10.1016/j.socscimed.2005.02.010 15970226 10.1016/j.socscimed.2005.02.010

[30] Stafford M, Marmot M (2003) Neighbourhood deprivation and health: does it affect us all equally? Int J Epidemiol 32:357–366. 10.1093/ije/dyg084 12777420 10.1093/ije/dyg084

[31] Chen X, Woo J, Yu R, Chung GK‑K, Yao W, Yeoh E‑K (2022) Subjective social status, area deprivation, and gender differences in health among Chinese older people. Int J Environ Res Public Health 19:9857. 10.3390/ijerph19169857 36011511 10.3390/ijerph19169857 PMC9408352

[32] Riva M, Bambra C, Curtis S, Gauvin L (2011) Collective resources or local social inequalities? Examining the social determinants of mental health in rural areas. Eur J Public Health 21:197–203. 10.1093/eurpub/ckq064 20484149 10.1093/eurpub/ckq064

[33] Allen J, Born S, Damerow S et al (2021) Gesundheit in Deutschland aktuell (GEDA 2019/2020-EHIS)-Hintergrund und Methodik. J Health Monit 6:72–87. 10.25646/8558

[34] American Psychiatric Association (1994) Diagnostic and statistical manual of mental disorders. Washington D.C

[35] Brauns H, Scherer S, Steinmann S (2003) The CASMIN educational classification in international comparative research. In: Advances in cross-national comparison: a European working book for demographic and socio-economic variables. Springer, S 221–244

[36] Eurostat (2024) Glossary: Equivalised income. In: Eurostat (ed) Statistics Explained. https://ec.europa.eu/eurostat/statistics-explained/index.php?title=Glossary:Equivalised_income. Zugegriffen: 16. Jan. 2025

[37] Bundesinstitut für Bau- Stadt- und Raumforschung (BBSR) (2018) INKAR – Indikatoren und Karten zur Raum- und Stadtentwicklung. https://www.inkar.de/. Zugegriffen: 16. Jan. 2025

[38] Vogt M (2009) Small Area Estimation: Die Schätzer von Fay-Herriot und Battese-Harter-Fulle. In: Statistisches Bundesamt (ed) Wirtschaft und Statistik. Statistisches Bundesamt. https://www.destatis.de/DE/Methoden/WISTA-Wirtschaft-und-Statistik/2009/02/diplomarbeit-small-area-estimation_022009.pdf?__blob=publicationFile. Zugegriffen: 16. Jan. 2025

[39] Getis A (2009) Spatial autocorrelation. In: Handbook of applied spatial analysis: software tools, methods and applications. Springer, S 255–278

[40] Rabe-Hesketh S, Skrondal A (2022) Multilevel and longitudinal modeling using Stata volume II: categorical responses, counts, and survival. Stata Press, College Station

[41] Woo J, Tang N, Suen E, Leung J, Wong M (2009) Green space, psychological restoration, and telomere length. Lancet 373:299–300. https://doi.org/10.1016/S0140–6736(09)60094‑5 19167568 10.1016/S0140-6736(09)60094-5

[42] Hunter MR, Gillespie BW, Chen SY‑P (2019) Urban nature experiences reduce stress in the context of daily life based on salivary biomarkers. Front Psychol 10:413490. 10.3389/fpsyg.2019.00722 10.3389/fpsyg.2019.00722 PMC6458297 31019479

[43] Flacke J, Schüle SA, Köckler H, Bolte G (2016) Mapping environmental inequalities relevant for health for informing urban planning interventions—A case study in the city of Dortmund, Germany. Int J Environ Res Public Health 13:711. 10.3390/ijerph13070711 27420090 10.3390/ijerph13070711 PMC4962252

[44] Mitchell G, Norman P, Mullin K (2015) Who benefits from environmental policy? An environmental justice analysis of air quality change in Britain, 2001–2011. Environ Res Lett 10:105009

[45] Milojevic A, Niedzwiedz CL, Pearce J et al (2017) Socioeconomic and urban-rural differentials in exposure to air pollution and mortality burden in England. Environ Health 16:1–10. 10.1186/s12940-017-0314-5 28985761 10.1186/s12940-017-0314-5 PMC6389046

[46] Lee S, Chung JH (2024) Association between perceived noise pollution and sleep quality: findings from the 2018 community health survey. Noise Health 26:346–353. 10.4103/nah.nah_42_24 39345075 10.4103/nah.nah_42_24 PMC11539994

[47] Buoli M, Grassi S, Caldiroli A et al (2018) Is there a link between air pollution and mental disorders? Environ Int 118:154–168. 10.1016/j.envint.2018.05.044 29883762 10.1016/j.envint.2018.05.044

[48] Steinmetz-Wood M, Wasfi R, Parker G, Bornstein L, Caron J, Kestens Y (2017) Is gentrification all bad? Positive association between gentrification and individual’s perceived neighborhood collective efficacy in Montreal, Canada. Int J Health Geogr 16:1–8. 10.1186/s12942-017-0096-6 28709431 10.1186/s12942-017-0096-6 PMC5513321

[49] Sampson RJ (2003) The neighborhood context of well-being. Perspect Biol Med 46:S53–S64. 10.1353/pbm.2003.0073 14563074

[50] McElroy E, McIntyre JC, Bentall RP et al (2019) Mental health, deprivation, and the neighborhood social environment: a network analysis. Clin Psychol Sci 7:719–734. 10.1177/2167702619830640

[51] Sund ER, Jørgensen SH, Jones A, Krokstad S, Heggdal M (2007) The influence of social capital on self-rated health and depression—The Nord-Trøndelag health study (HUNT). Nor Epidemiol. 10.5324/nje.v17i1.173

[52] Drösler S, Greiner W, Läer S et al (2024) Gutachten zu den Wirkungen der regionalen Merkmale im Risikostrukturausgleich. In: Wissenschaftlicher Beirat zur Weiterentwicklung des Risikostrukturausgleichs beim Bundesamt für Soziale Sicherung, Bd. 16. Bundesamt für Soziale Sicherung, Bonn

