# Supplementary material for: Area-level socioeconomic deprivation, socioeconomic status, and depressive symptoms: a multilevel analysis using data from the GEDA 2019/2020 study
Source: Bundesgesundheitsblatt Gesundheitsforschung Gesundheitsschutz. 2025 Sep 9;68(10):1118–27. [Article in German] doi: 10.1007/s00103-025-04123-4 (PMC12460575; doi:10.1007/s00103-025-04123-4)
Supplement: Supplementary file 1 — Das Onlinematerial enthält ergänzende Ergebnisse zur Studie, unter anderem die auf Kreisebene geschätzten Prävalenzen depressiver Symptomatik, getrennt nach Geschlecht. Darüber hinaus werden die Ergebnisse der Mehrebenenregressionen mit vollständigen Kovariaten dargestellt, sowohl für die Gesamtstichprobe als auch getrennt nach Geschlecht. [file 103_2025_4123_MOESM1_ESM.pdf]

Onlinematerial

Regionale sozioökonomische Deprivation, sozioökonomischer Status und depressive Symptomatik: eine Mehrebenenanalyse mit Daten der Studie GEDA 2019/2020

Lina Wollgast[1,2], Christina Kersjes[1], Claudia Hövener[1,3], Niels Michalski[1]

[1] Robert Koch-Institut, Abteilung für Epidemiologie und Gesundheitsmonitoring, Berlin, Deutschland

[2] Humboldt-Universität zu Berlin, Institut für Sozialwissenschaften, Berlin, Deutschland

[3] Alice Salomon Hochschule Berlin, Fachbereich I: Soziale Arbeit, Berlin, Deutschland

Abb. A1 Geschätzte Prävalenz depressiver Symptomatik bei Frauen

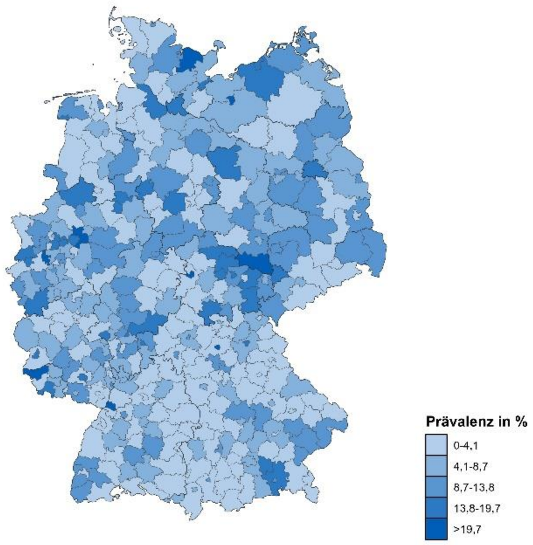

Morans I Frauen mit Kontextmerkmalen: 0,07 ($p < 0,01$)

Morans I Frauen ohne Kontextmerkmale: 0,04 ($p = 0,02$)

Abb. A2 Geschätzte Prävalenz depressiver Symptomatik bei Männern

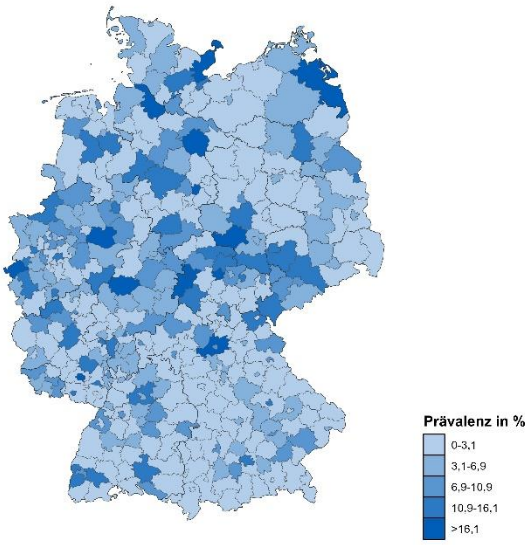

Morans I Männer mit Kontextmerkmalen: 0,04 ($p = 0,02$)

Morans I Männer ohne Kontextmerkmale: 0,01 ($p = 0,2$)

Tab. A1 Ergebnisse der logistischen Mehrebenenmodelle mit vollständigen Kovariaten unter Angabe von Odds Ratios, 95% Konfidenzintervallen und p-Werten

	(1)	(2)	(3)	(4)	(5)	(6)
Geschlecht (Ref. Männlich)						
Weiblich		1,32*	1,33*	1,29*	1,28*	1,29*
		[1,06;1,66]	[1,06;1,66]	[1,02;1,63]	[1,02;1,62]	[1,02;1,64]
Alter (Ref. 18-29)						
30-44 Y.		0,76	0,76	0,97	0,98	0,96
		[0,50;1,16]	[0,50;1,16]	[0,63;1,47]	[0,65;1,49]	[0,63;1,47]
45-59 Y.		1,00	1,00	1,13	1,15	1,13
		[0,72;1,39]	[0,72;1,39]	[0,81;1,58]	[0,82;1,60]	[0,81;1,59]
60-79 Y.		0,43***	0,43***	0,43***	0,43***	0,43***
		[0,30;0,61]	[0,30;0,61]	[0,30;0,62]	[0,30;0,62]	[0,30;0,62]
80+		0,47***	0,47***	0,42***	0,42***	0,42***
		[0,31;0,72]	[0,30;0,72]	[0,26;0,67]	[0,26;0,67]	[0,26;0,67]
Migrationshintergrund (Ref. Nein)						
Ja		1,61***	1,61***	1,47**	1,47**	1,47**
		[1,24;2,07]	[1,25;2,08]	[1,12;1,92]	[1,12;1,92]	[1,12;1,93]
Chronische Vorerkrankungen (Ref. Keine)						
Mind. 1		4,13***	4,09***	3,78***	3,81***	3,78***
		[2,98;5,72]	[2,96;5,66]	[2,73;5,23]	[2,76;5,25]	[2,73;5,23]
Wohnort (Ref. Westdeutschland)						
Ostdeutschland (inkl. Berlin)		1,51*	1,12	1,20	1,22	1,20
		[1,08;2,10]	[0,79;1,57]	[0,84;1,70]	[0,85;1,74]	[0,84;1,70]
Siedlungsstruktureller Kreistyp (Ref. Kreisfreie Großstädte)						
Städtische Kreise		0,50***	0,56***	0,59**	0,59**	0,59**
		[0,36;0,69]	[0,39;0,78]	[0,42;0,84]	[0,42;0,83]	[0,42;0,84]
Ländliche Kreise		0,48***	0,48***	0,49***	0,48***	0,49***
		[0,33;0,70]	[0,33;0,70]	[0,33;0,72]	[0,33;0,72]	[0.33;0,72]
Dünn besiedelte ländliche Kreise		0,33***	0,30***	0,31***	0,30***	0,31***
		[0,22;0,50]	[0,20;0,46]	[0,20;0,47]	[0,20;0,46]	[0,20;0,47]
Erhebungszeitpunkt (Ref. Vor dem 15.03.2020)						
Ab dem 15.03.2020		0,75**	0,74**	0,77*	0,77*	0,77*
		[0,60;0,93]	[0,59;0,92]	[0,62;0,97]	[0,61;0,96]	[0,62;0,97]
Regionale sozioökonomische Deprivation (Ref. Niedrige Deprivation)						
Mittlere Deprivation			1,86***	1,74***	1,23	1,78^{+}
			[1,37;2,53]	[1,27;2,38]	[0,75;2,00]	[0,99;3,18]
Hohe Deprivation			3,29***	2,80***	2,36**	3,50**
			[2,14;5,07]	[1,82;4,30]	[1,26;4,39]	[1,44;8,52]
Bildung (Ref. Hohe Bildung)						
Niedrige Bildung				2,56***	1,76*	2,55***
				[1,84;3,56]	[1,12;2,76]	[1,83;3,55]
Mittlere Bildung				1,73***	1,52*	1,72***
				[1,36;2,20]	[1,00;2,31]	[1,35;2,19]
Einkommen (Ref. >150% des Medians)						
<60% des Medians				3,57***	3,58***	3,93***
				[2,52;5,05]	[2,53;5,07]	[2,29;6,75]
60% - 150% des Medians				1,86***	1,86***	1,94**
				[1,39;2,49]	[1,39;2,49]	[1,19;3,15]
Niedrige Bildung * Mittlere Deprivation					2,01*	
					[1,11;3,64]	
Niedrige Bildung * Hohe Deprivation					1,19	
					[0,55;2,56]	
Mittlere Bildung * Mittlere Deprivation					1,21	
					[0,72;2,04]	
Mittlere Bildung * Hohe Deprivation					1,22	
					[0,64;2,34]	
<60% * Mittlere Deprivation						0,85
						[0,42;1,68]
<60% * Hohe Deprivation						0,91
						[0,34;2,44]
60% - 150% * Mittlere Deprivation						1,05
						[0,57;1,92]
60% - 150% * Hohe Deprivation						0,70
						[0,27;1,79]
Random Intercept	3,10	3,14	3,08	3,03	3,06	3,04
(Gemeindeebene)	[2,54;3,80]	[2,56;3,86]	[2,50;3,79]	[2,47;3,71]	[2,49;3,75]	[2,47;3,73]
Intraklassen-Korrelation	0,486	0,489	0,484	0,479	0,482	0,480
N (Gemeinden)	2.068	2.068	2.068	2.068	2.068	2.068
N (Individuen)	18.362	18.362	18.362	18.362	18.362	18.362

$^{+}$ $p < 0,1$, * $p < 0,05$, ** $p < 0,01$, *** $p < 0,001$

Tab. A2 Ergebnisse der logistischen Mehrebenenmodelle für Frauen mit vollständigen Kovariaten unter Angabe von Odds Ratios, 95% Konfidenzintervallen und p-Werten

	(1)	(2)	(3)	(4)	(5)	(6)
Alter (Ref. 18-29)						
30-44 Y.		0,63	0,62	0,77	0,79	0,76
		[0,32;1,21]	[0,32;1,20]	[0,40;1,49]	[0,41;1,52]	[0,39;1,49]
45-59 Y.		0,72	0,72	0,81	0,82	0,81
		[0,42;1,23]	[0,42;1,23]	[0,48;1,38]	[0,48;1,39]	[0,48;1,37]
60-79 Y.		$0,29^{***}$	$0,29^{***}$	$0,29^{***}$	$0,29^{***}$	$0,29^{***}$
		[0,17;0,50]	[0,17;0,50]	[0,17;0,48]	[0,17;0,49]	[0,17;0,48]
80+		$0,28^{***}$	$0,28^{***}$	$0,24^{***}$	$0,24^{***}$	$0,24^{***}$
		[0,14;0,54]	[0,14;0,54]	[0,12;0,47]	[0,13;0,47]	[0,13;0,47]
Migrationshintergrund (Ref. Nein)						
Ja		1,27	1,26	1,19	1,22	1,20
		[0,90;1,78]	[0,90;1,77]	[0,84;1,69]	[0,86;1,72]	[0,84;1,71]
Chronische Vorerkrankungen (Ref. Keine)						
Mind. 1		$6,92^{***}$	$6,88^{***}$	$6,52^{***}$	$6,60^{***}$	$6,56^{***}$
		[3,95;12,11]	[3,93;12,04]	[3,69;11,51]	[3,74;11,67]	[3,71;11,60]
Wohnort (Ref. Westdeutschland)						
Ostdeutschland (inkl. Berlin)		$1,80^{**}$	1,43	$1,57^{+}$	$1,56^{+}$	$1,54^{+}$
		[1,17;2,79]	[0,91;2,23]	[0,99;2,49]	[0,98;2,49]	[0,97;2,45]
Siedlungsstruktureller Kreistyp (Ref. Kreisfreie Großstädte)						
Städtische Kreise		$0,54^{**}$	$0,60^{*}$	$0,62^{*}$	$0,61^{*}$	$0,63^{*}$
		[0,36;0,83]	[0,39;0,91]	[0,40;0,96]	[0,39;0,94]	[0,41;0,98]
Ländliche Kreise		$0,58^{*}$	$0,59^{*}$	$0,59^{*}$	$0,57^{*}$	$0,60^{*}$
		[0,36;0,93]	[0,36;0,95]	[0,36;0,96]	[0,35;0,94]	[0,36;0,99]
Dünn besiedelte ländliche Kreise		$0,25^{***}$	$0,23^{***}$	$0,24^{***}$	$0,22^{***}$	$0,24^{***}$
		[0,14;0,42]	[0,14;0,40]	[0,13;0,41]	[0,13;0,39]	[0,14;0,42]
Erhebungszeitpunkt (Vor dem 15.03.2020)						
Ab dem 15.03.2020		$0,76^{*}$	$0,75^{*}$	$0,79^{+}$	$0,78^{+}$	0,80
		[0,58;0,99]	[0,58;0,98]	[0,61;1,03]	[0,60;1,01]	[0,62;1,04]
Regionale sozioökonomische Deprivation (Ref. Niedrige Deprivation)						
Mittlere Deprivation			$1,53^{*}$	1,39	1,00	1,47
			[1,02;2,30]	[0,92;2,10]	[0,52;1,93]	[0,72;3,02]
Hohe Deprivation			$2,39^{***}$	$1,99^{**}$	$2,10^{+}$	$4,09^{*}$
			[1,45;3,95]	[1,20;3,31]	[0,92;4,82]	[1,24;13,47]
Bildung (Ref. Hohe Bildung)						
Niedrige Bildung				$2,29^{**}$	$1,75^{+}$	$2,26^{**}$
				[1,33;3,92]	[0,95;3,24]	[1,32;3,87]
Mittlere Bildung				$1,73^{**}$	1,51	$1,72^{**}$
				[1,21;2,48]	[0,92;2,49]	[1,21;2,46]
Einkommen (Ref. >150% des Medians)						
<60% des Medians				$3,14^{***}$	$3,15^{***}$	$4,81^{***}$
				[1,92;5,12]	[1,94;5,11]	[2,51;9,24]
60% - 150% des Medians				$1,45^{+}$	$1,45^{+}$	1,48
				[0,98;2,15]	[0,99;2,14]	[0,84;2,59]
Niedrige Bildung * Mittlere Deprivation					2,00	
					[0,85;4,72]	
Niedrige Bildung * Hohe Deprivation					0,59	
					[0,19;1,83]	
Mittlere Bildung * Mittlere Deprivation					1,24	
					[0,63;2,44]	
Mittlere Bildung * Hohe Deprivation					1,14	
					[0,46;2,84]	
<60% * Mittlere Deprivation						0.57
						[0.25,1.34]
<60% * Hohe Deprivation						0.44
						[0.11,1.78]
60% - 150% * Mittlere Deprivation						1.21
						[0.56,2.58]
60% - 150% * Hohe Deprivation						0.43
						[0.12,1.55]
Random Intercept	3,64	3,64	3,60	3,63	3,65	3,66
(Gemeindeebene)	[2,79;4,74]	[2,78;4,76]	[2,74;4,72]	[2,77;4,74]	[2,79;4,76]	[2,81;4,78]
Intraklassen-Korrelation	0,525	0,525	0,522	0,524	0,526	0,527
N (Gemeinden)	1.679	1.679	1.679	1.679	1.679	1.679
N (Individuen)	9.502	9.502	9.502	9.502	9.502	9.502

$^{+}$ $p < 0,1$, * $p < 0,05$, ** $p < 0,01$, *** $p < 0,001$

Tab. A3 Ergebnisse der logistischen Mehrebenenmodelle für Männer mit vollständigen Kovariaten unter Angabe von Odds Ratios, 95% Konfidenzintervallen und p-Werten

	(1)	(2)	(3)	(4)	(5)	(6)
Alter (Ref. 18-29)						
30-44 Y.		0,90	0,90	1,30	1,31	1,28
		[0,51;1,58]	[0,51;1,58]	[0,75;2,27]	[0,75;2,29]	[0,74;2,23]
45-59 Y.		1,49	1,49	1,87*	1,90*	1,87*
		[0,90;2,46]	[0,90;2,46]	[1,12;3,13]	[1,14;3,17]	[1,12;3,13]
60-79 Y.		0,66	0,66	0,77	0,77	0,77
		[0,36;1,22]	[0,36;1,21]	[0,43;1,39]	[0,43;1,39]	[0,43;1,38]
80+		0,87	0,85	1,06	1,04	1,05
		[0,43;1,74]	[0,43;1,70]	[0,50;2,24]	[0,48;2,25]	[0,50;2,22]
Migrationshintergrund (Ref. Nein)						
Ja		2,14**	2,15**	1,89*	1,85*	1,90*
		[1,35;3,39]	[1,36;3,41]	[1,16;3,08]	[1,14;3,02]	[1,16;3,10]
Chronische Vorerkrankungen (Ref. Keine)						
Mind. 1		2,89***	2,86***	2,59***	2,62***	2,60***
		[1,78;4,68]	[1,78;4,61]	[1,60;4,21]	[1,62;4,21]	[1,61;4,20]
Wohnort (Ref. Westdeutschland)						
Ostdeutschland (inkl. Berlin)		1,20	0,85	0,84	0,88	0,85
		[0,71;2,02]	[0,49;1,47]	[0,48;1,48]	[0,50;1,56]	[0,48;1,49]
SiedlungsstrukturellerKreistyp (Ref. Kreisfreie Großstädte)						
Städtische Kreise		0,33***	0,35***	0,39***	0,39***	0,38***
		[0,21;0,51]	[0,22;0,56]	[0,24;0,63]	[0,24;0,63]	[0,23;0,62]
Ländliche Kreise		0,31***	0,28***	0,30***	0,30***	0,29***
		[0,18;0,53]	[0,16;0,50]	[0,17;0,53]	[0,17;0.53]	[0,16;0,52]
Dünn besiedelte ländliche Kreise		0,41**	0,33***	0,37***	0,36***	0,36***
		[0,24;0,70]	[0,19;0,59]	[0,20;0,66]	[0,20;0,65]	[0,20;0,65]
Erhebungszeitpunkt (Ref. Vor dem 15.03.2020)						
Ab dem 15.03.2020		0,79	0,78	0,83	0,82	0,82
		[0,53;1,17]	[0,53;1,16]	[0,54;1,26]	[0,54;1,24]	[0,54;1,25]
Regionale sozioökonomische Deprivation (Ref. Niedrige Deprivation)						
Mittlere Deprivation			2,56***	2,39***	1,93^{+}	2,18
			[1,59;4,12]	[1,48;3,88]	[0,92;4,05]	[0,84;5,66]
Hohe Deprivation			4,63***	3,76***	3,18*	3,13
			[2,38;9,00]	[2,00;7,06]	[1,12;9,01]	[0,77;12,75]
Bildung (Ref. Hohe Bildung)						
Niedrige Bildung				2,46***	1,58	2,46***
				[1,54;3,92]	[0,79;3,16]	[1,55;3,91]
Mittlere Bildung				1,92***	2,05*	1,91***
				[1,31;2,82]	[1,08;3,87]	[1,31;2,80]
HH-Einkommen (Ref. >150% des Medians)						
<60% des Medians				5,56***	5,62***	3,95**
				[3,13;9,89]	[3,16;10,01]	[1,49;10,44]
60% - 150% des Medians				2,69***	2,72***	2,82*
				[1,66;4,35]	[1,69;4,39]	[1,24;6,42]
Niedrige Bildung * Mittlere Deprivation					2,00	
					[0,73;5,48]	
Niedrige Bildung * Hohe Deprivation					1,66	
					[0,48;5,71]	
Mittlere Bildung * Mittlere Deprivation					0,92	
					[0,41;2,04]	
Mittlere Bildung * Hohe Deprivation					0,92	
					[0,28;3,01]	
<60% * Mittlere Deprivation						1,48
						[0,45;4,88]
<60% * Hohe Deprivation						2,13
						[0,37;12,28]
60% - 150% * Mittlere Deprivation						0,99
						[0,35;2,76]
60% - 150% * Hohe Deprivation						0,92
						[0,22;3,88]
Random Intercept	4,97	5,02	4,91	4,74	4,77	4,79
(Gemeindeebene)	[3,71;6,65]	[3,79;6,65]	[3,72;6,47]	[3,63;6,18]	[3,66;6,23]	[3,66;6,28]
Intraklassen-Korrelation	0,602	0,604	0,599	0,590	0,592	0,593
N (Gemeinden)	1.688	1.688	1.688	1.688	1.688	1.688
N (Individuen)	8.860	8.860	8.860	8.860	8.860	8.860

$^{+}$ $p < 0,1$, * $p < 0,05$, ** $p < 0,01$, *** $p < 0,001$
